# Subnational certification of elimination of mother-to-child transmission of HIV and/or syphilis: a Brazilian experience report

**DOI:** 10.1590/S2237-96222023000300003.EN

**Published:** 2023-10-30

**Authors:** Angélica Espinosa Miranda, Pâmela Cristina Gaspar, Leonor Henriette de Lannoy, Aranaí Sampaio Diniz Guarabyra, Rayone Moreira Costa Veloso Souto, Esdras Daniel dos Santos Pereira, Gerson Fernando Pereira, Guilherme Borges Dias, Carmen Silvia Bruniera Domingues, Aparecida Morais Lima, Ariane Tiago Bernardo de Matos, Maria da Guia de Oliveira, Mayra Gonçalves Aragón, Nádia Maria da Silva Machado, Luíz Fernando Aires, Isabella Mayara Diana de Souza, Ethel Leonor Maciel, Draurio Barreira

**Affiliations:** 1 Ministério da Saúde, Departamento de HIV/Aids, Tuberculose, Hepatites Virais e Infecções Sexualmente Transmissíveis, Brasília, DF, Brazil; 2 Ministério da Saúde, Secretaria de Vigilância em Saúde e Ambiente, Brasília, DF, Brazil

**Keywords:** Mother-to-child Transmission, HIV, Syphilis, Public Health Surveillance, Certification, Transmissão Vertical, HIV, Sífilis, Vigilância em Saúde Pública, Certificação, Trasmisión Vertical, VIH, Sífilis, Vigilancia en Salud Pública, Certificación

## Abstract

**Objective:**

to describe the subnational implementation process of the certification for elimination of mother-to-child transmission of HIV and/or syphilis, its main barriers, challenges and opportunities.

**Methods:**

in 2022, indicators from the last full year for impact targets and the last two full years for process targets, available in national information systems, were evaluated; descriptive reports were analyzed and actions were acknowledged within four thematic axes, according to PAHO/WHO recommendations.

**Results:**

43 municipalities ≥ 100,000 inhabitants were certified, covering 24.6 million inhabitants; one municipality achieved dual elimination (HIV-syphilis), 28 municipalities achieved elimination of HIV and 10 received silver tiers; regarding syphilis, one elimination was observed, along with 4 gold tiers, 13 silver tiers and 4 bronze tiers; a higher number of certifications was identified in the Southeast and South regions.

**Conclusion:**

barriers and challenges of the process were overcome through tripartite collaboration; the experience provided better integration of surveillance with care and improved actions aimed at preventing mother-to-child transmission.

## Study contributions

**Main results:**

First experience of the sub-national process of certification of elimination of mother-to-child transmission (MTCT) of HIV and/or syphilis at a global level. In 2022, 43 municipalities ≥ 100,000 inhabitants were certified, covering 24.6 million inhabitants.

**Implications for services:**

The experience of sub-national certification of the EMTCT was important in mobilizing the municipalities that engaged in its initiatives, worked to improve the quality of care and surveillance and emerging as the main proponents in the process.

**Perspectives:**

Through this ongoing and dynamic initiative, there is an anticipation of over 100 municipalities and states joining in 2023. Sub-national certification aims to enhance comprehensive care for pregnant women, in order to achieve national certification of EMTCT.

## Resumen

**Objetivo:**

describir el proceso de implementación subnacional de la certificación de eliminación de la transmisión vertical (TV) de sífilis y/o VIH, barreras, oportunidades y desafíos.

**Métodos:**

en 2022, se evaluaron indicadores del último año completo para la meta de impacto y de los dos últimos años para las de proceso en los sistemas de información; se analizaron informes descriptivos y se reconocieron acciones de cuatro ejes, según las recomendaciones de la OPS/OMS.

**Resultados:**

se certificaron 43 municipios ≥ 100.000 mil habitantes, cubriendo 24,6 millones de habitantes; un municipio logró la doble eliminación (VIH-sífilis), 28 la eliminación del VIH y 10 sellos plata; para sífilis, hubo una eliminación, 4 sellos oro, 13 plata y 4 bronce; las regiones Sudeste y Sur obtuvieron más certificaciones.

**Conclusión:**

barreras y desafíos fueron superados mediante la colaboración tripartita; la experiencia permitió la integración de la vigilancia con la atención y la cualificación de acciones para la prevención de la TV.

## Resumo

**Objetivo:**

descrever o processo de implantação subnacional da certificação da eliminação da transmissão vertical de HIV e/ou sífilis, suas principais barreiras, desafios e oportunidades.

**Métodos:**

em 2022, foram avaliados indicadores do último ano completo para meta de impacto, e dos dois últimos anos completos para metas de processo, disponíveis nos sistemas nacionais de informações; foram analisados relatórios descritivos e reconhecidas ações em quatro eixos temáticos, conforme recomendações da OPAS/OMS.

**Resultados:**

43 municípios ≥ 100 mil habitantes foram certificados, abrangendo 24,6 milhões de habitantes; um município alcançou dupla eliminação (HIV-sífilis), 28 alcançaram eliminação para HIV e 10, selos prata; para sífilis, houve uma eliminação, 4 selos ouro, 13 prata e 4 bronze; identificou-se maior número de certificações nas regiões Sudeste e Sul.

**Conclusão:**

barreiras e desafios do processo foram superados pela colaboração tripartite; a experiência proporcionou melhor integração da vigilância com a assistência e qualificação das ações para prevenção da transmissão vertical.

## INTRODUCTION

Despite the technological advancements in recent decades, mother-to-child transmission (MTCT) of syphilis and the human immunodeficiency virus, (HIV), remains a major public health problem, given its impacts on maternal and child health.^1^ ^),(^ ^2^ MTCT prevention is one of the strategies established to achieve the 2030 Agenda for Sustainable Development Goals adopted by the United Nations, aiming to promote health and well-being, ensure human rights, achieve gender equality and reduce inequalities.^3^ ^),(^ ^4^

Since the 1990s, the Pan American Health Organization (PAHO) has encouraged the reduction of congenital syphilis incidence to less than 0.5 cases per 1,000 live births. Several recommendations and guidelines have been published, and a plan has been proposed for the Member States to promote the elimination of mother-to-child transmission (EMTCT) of HIV and syphilis in the Americas, with targets set to be met by 2015.^5^ Cuba was the first country in the world to receive certification from WHO that it has eliminated MTCT of HIV and syphilis.^6^ PAHO’s initial plan was expanded in 2016 to include hepatitis B and Chagas disease.^7^

Following PAHO’s initiative, the World Health Organization (WHO), in 2014, released the Global guidance on the criteria and processes for certification: EMTCT of HIV and/or syphilis. These criteria were expanded in 2017, when it was established the possibility for countries with a high prevalence of HIV and/or syphilis to be certified for the bronze, silver or gold tiers in “the path to elimination”, encouraging gradual reduction in MTCT rates.^8^

In the same year, 2017, Brazil launched the first subnational guide on EMTCT of HIV, with the municipality of Curitiba receiving certification; in 2019, the municipalities of São Paulo and Umuarama were certified. In 2021, the Brazilian guide was updated and included MTCT of syphilis; in addition, a second edition of the document, based on the global guidance proposed by the WHO, enabled single (HIV or syphilis) and dual certification, in addition to providing for certification for gold, silver and bronze tiers on the Path to Elimination of MTCT of HIV and/or syphilis, in order to encompass places that have not achieved the elimination targets but show indicators that are closer to (Box 1).^8^ National policy adopted subnational certification for EMTCT for municipalities with 100,000 inhabitants or over,^9^ raising awareness of the topic and discussing the importance of integrated, high-quality surveillance and care.^10^ ^),(^ ^11^

The update of the guide invigorated the subnational certification process by including the tiers of progress towards elimination. This measure was fundamental to meet Brazilian context: significant regional disparities and a high detection rate of syphilis during pregnancy. Despite the progress in prenatal care coverage across the country, the quality of services offering timely intervention and treatment, especially for syphilis, was not sufficient to contain MTCT.^12^ ^),(^ ^13^ Studies indicate that prenatal testing failures, inadequate or lack of treatment for syphilis and HIV in pregnant women account for the majority of cases of congenital syphilis and MTCT of HIV.^1^ ^),(^ ^14^ ^),(^ ^15^

The achievement of the subnational certification process for the elimination of HIV and/or syphilis, or the tiers of progress toward elimination, was based on the performance of municipal impact and process indicators.^8^ ^),(^ ^9^

This article aimed to describe the implementation process of the certification of elimination of MTCT of HIV and/or syphilis in Brazil, its main barriers, challenges and opportunities.

## METHODS

The certification process of EMTCT of HIV and/or syphilis occurred in municipalities with ≥100,000 inhabitants during 2022, as a partnership strategy involving the Ministry of Health, states and municipalities, aiming to improve actions for reducing MTCT of HIV and syphilis, infections still considered a public health problem in the country.^9^ ^)^ The years evaluated were 2019 and 2020.

**Figure 1:**
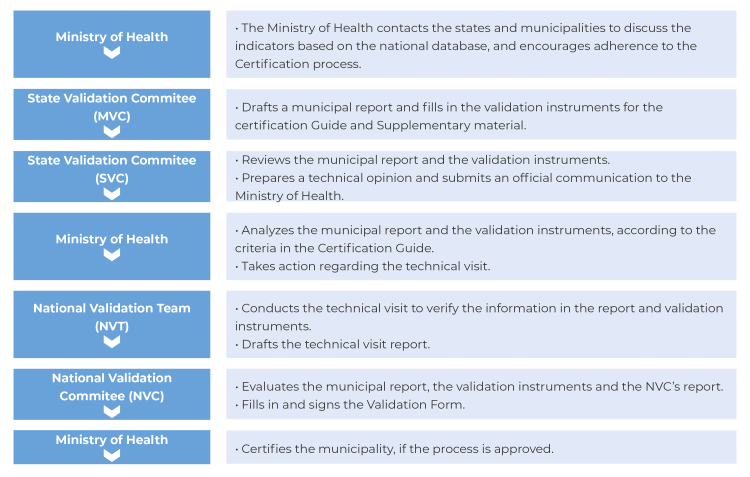
- Operational flow of the municipal certifications process of mother-to-child transmission of HIV^a^ and/or syphilis based on the responsibilities of the Ministry of Health, committees and validation team

The steps for implementing the certification were as follows: (i) the establishment of the National Validation Committee for MTCT; (ii) definition of criteria and process and impact indicators; (iii) development of a list of cities with ≥ 100,000 inhabitants and eligible states, meeting the recommended indicators; (iv) proposal presentation to states and municipalities; (v) standardized development process of the certification report, based on the supplement to the Guide for Certification of Elimination of Mother-to-child Transmission of HIV and/or Syphilis, by the candidate municipality or state; (vi) analysis of municipal reports by the Ministry of Health; (vii) establishment and training of the National Validation Team (NVT), comprised of experts from each thematic axis; (viii) development of the field manual for the NVT; (ix) planning and performance of on-site visits by the NVT, based on the supplement to the Guide;^16^ (x) validation by the National Certification Committee (NVC); and finally, (xi) certification formalized through a public event, involving awarding of plaques and certificates. The operationalization of the process commenced with the establishment of certification request and grant workflow (Figure 1).

a) HIV: Human Immunodeficiency Virus.

Note: The flow of requests for and granting of certification begins with an initial contact by the Ministry of Health with states and municipalities; this is followed by the drafting of a report by the local municipal team, based on the Supplement to the Certification Guide;^16^ after review and issuance of an opinion by the state, the same report is forwarded to the Ministry of Health; the Ministry of Health then conducts a comprehensive analysis of the document, regarding compliance with minimum criteria. If these are met, it proceeds to the on-site visit, carried out by the National Validation Team (NVT); upon conclusion of this stage, the NVT compiles a report of the visit, which is then submitted for appraisal of the National Validation Committee (NVC), comprised of representatives from various institutions related to the subject; finally, the NVC decides whether or not to approve the certification.

In the Guide for Certification of Elimination of Mother-to-child Transmission of HIV and/or Syphilis, the subnational certification strategy acknowledges the development of actions distributed in four thematic axes, according to PAHO/WHO recommendations:

a) programs and services;

b)epidemiological surveillance and data quality;

c) diagnostic capacity and test quality; and

d)human rights, gender equality and community participation.

These four axes were structured to induce organization and improvement of care based on the best evidence, according to the logistical and technological resources available.

This spans from early prenatal care with timely access to testing and appropriate treatment, to surveillance, through case monitoring and control.^9^

The requirements and minimum criteria for municipality certification included (i) prior achievement of impact and process indicators, established in the Guide for Certification of EMTCT (Box 1), (ii) the implementation of Investigation Committee for MTCT of HIV and/or Syphilis, or technical/working groups or the Maternal and Infant Mortality Committee, (iii) availability of a surveillance and monitoring system for cases of MTCT of HIV and/or syphilis, (iv) evidence of appropriate preventive measures for EMTCT of HIV and/or syphilis, and (v) protection of fundamental human rights, including the right to health and its social determinants.^9^

**Box 1:** Impact and process targets for certification of elimination of mother-to-child transmission of HIV^a^ and/or syphilis, Brazil

Minimum criteria	Parameters			
	Elimination	Tier		
Impact target^b^		Gold	Silver	Bronze
1) Incidence rate of HIV-infected children due to mother-to-child transmission^c^	≤ 0.5 case per 1,000 LBs^d^	≤ 1.0 case per 1,000 LBs^d^	≤ 1.5 case per 1,000 LBs^d^	≤ 2.0 cases per 1,000 LBs^d^
2) Rate of mother-to-child transmission of HIV^c^	≤ 2%	≤ 2%	≤ 2%	≤ 2%
3) Incidence rate of congenital syphilis	≤ 0.5 case per 1,000 LBs^d^	≤ 2.5 cases per 1,000 LBs^d^	≤ 5.0 cases per 1,000 LBs^d^	≤ 7.5 cases per 1,000 LBs^d^
Process targets^a^	Elimination	Gold	Silver	Bronze
1) Minimum coverage of four prenatal care visits	≥ 95%	≥ 95%	≥ 90%	≥ 90%
2) Coverage for pregnant women who underwent at least one HIV^c^ test during prenatal care				
3) Coverage for pregnant women living with HIV^c^ using antiretroviral therapy during prenatal care				
4) Coverage for pregnant women who underwent at least one syphilis test during prenatal care				
5) Coverage for pregnant women adequately treated for syphilis during prenatal care				

a) HIV: Human Immunodeficiency Virus; b) For impact targets, the last full year of each disease was taken into consideration, and for process targets, we took into consideration the last two full years, given the need to complete the investigation of the case; c) Rates adapted for Brazil, lower than those recommended by the Pan American Health Organization/World Health Organization (PAHO/WHO), given the low incidence of HIV in the country; c) d) LBs: Live births.

Source: Adapted from the Guide for certification of Elimination of Mother-to-Child Transmission.^9^

Of the total of 326 Brazilian municipalities with ≥ 100 thousand inhabitants, 85 were initially considered eligible based on the analysis of impact and process indicators and targets available in the national information systems; with the exception of testing, whose source is local. State administrators, contacted by the Ministry of Health via e-mail and phone calls, were asked to participate in the certification process by crafting strategies to support municipal administrators and health professionals of the network, aiming at epidemiological analysis of the data and development of the descriptive report.

The main stage of the process was the on-site visit to the municipalities, carried out by the NVT, whose members should come from different states than those of the municipalities visited. The purpose of these visits was to validate and enhance the information provided in the report submitted by the municipality. This activity was divided into two segments: first, a technical and political meeting with state and municipal administrators, health professionals and local non-governmental organizations (NGOs) involved in the process; and secondly, the visit to the health services that comprise the maternal and child health care network [surveillance, ombudsman office, laboratory, primary care centers, specialized care service (SCS), counseling and testing center (CTC), maternity hospital, Street Clinic, among others]. The primary care centers visited should preferably be located in regions of higher social vulnerability.

In order to standardize the visits, (i) a list of evaluation points was developed for each type of request (HIV, syphilis or dual certification), according to the four axes previously described, and (ii) a structured framework was developed for generating reports during technical visits, including the evaluated points and observations.^16^ During the visits, which lasted from 3 to 5 days, depending on the municipality’s size and the national team’s schedule, the journey of the pregnant woman was traced in collaboration with the local health team. This aimed to observe the organization of care, adequacy of procedures, access to services and surveillance actions. Individual interviews were conducted with a convenience sample involving healthcare professionals and service users (pregnant or postpartum women). Information systems and medical records were also verified.

A report was drafted for each visit in order to support the National Validation Committee in making the final judgment on the granting of certification. This Committee is comprised of representatives of the Ministry of Health, the National Council of Health Secretaries (*Conselho Nacional de Secretários de Saúde* - CONASS), the National Council of Municipal Health Secretaries (*Conselho Nacional de Secretarias Municipais de Saúd*e - CONASEMS), professional councils (medicine, nursing and pharmacy), scientific societies, PAHO, UNICEF, UNAIDS and the National Regulatory Agency for Private Health Insurance and Plans (*Agência Nacional de Saúde* - ANS). The visit report was also sent to the municipalities, as part of an ongoing process of improvement and qualification.

The subnational certification is valid for 2 years for municipalities ≥ 1 million inhabitants, and 3 years for municipalities with ≥ 100,000 and < 1 million. Throughout this period, municipalities are required to sustain their indicator performances annually, through available information systems.

The data analyzed are available in previously published reports, which is why the work was not submitted to ethical appraisal.

## RESULTS

**Figure 2:**
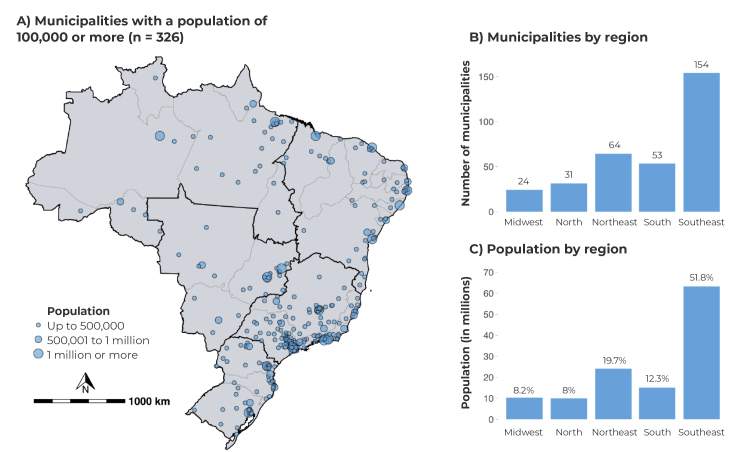
- Distribution of Brazilian municipalities with 100,000 inhabitants or more, according to population size (A) regions (B) and population per region (C)

The majority of the 326 municipalities with ≥ 100,000 inhabitants in Brazil are located in the Southeast region (n = 154), followed by the Northeast region (n = 64). These regions encompass 71.5% of the Brazilian population living in municipalities of this size (Figure 2).

In total, 45 certification requests were received from municipalities, across the country’s five macro-regions, and 43 received certification and/or tiers of progress toward elimination. The process involved visits to 337 health services within the maternal and child health care network. Services located in areas of greater social and individual vulnerability, as well as maternity hospitals and specialized care services (SCS) were given priority. Local specificities and schedule compliance represented challenges experienced by the NVT.

**Figure 3:**
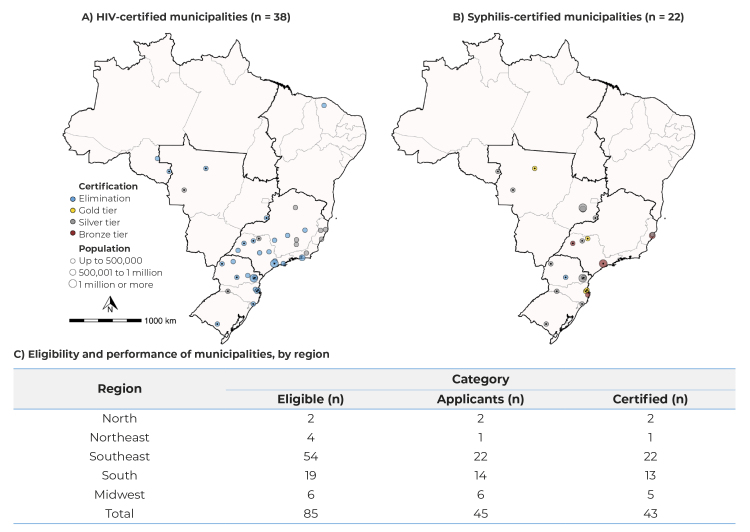
- Distribution of Brazilian municipalities, according to certification for the elimination or towards the elimination of mother-to-child transmission of HIV (A), syphilis (B) and eligibility for HIV and/or syphilis certification, by performance of process and outcome indicators (C)

Note: Two municipalities were not certified because they did not meet the criteria established during the analysis of the report sent by the municipality and the on-site visit.

At the end of the process, in 2022, of the 43 certifications awarded, 38 were for HIV and 22 for syphilis. One municipality achieved dual elimination of both HIV and syphilis. The highest number of certifications occurred in the Southeast and South regions of the country. The total number of municipalities that were certified encompassed 24.6 million inhabitants. Regarding the categories, 28 municipalities achieved elimination of HIV, while 10 received silver tiers. As for syphilis, there was one elimination, 4 gold, 13 silver and 4 bronze tiers (Figures 3A and 3B).

## DISCUSSION

The subnational certification process of EMTCT of HIV and/or syphilis in Brazil was the first experience in this regard, and can serve as a model for other countries. Implemented in 2022 for both health conditions, the initiative showed the importance of collaborating with states and municipalities in organizing service networks and surveillance of these infections.

Some barriers and challenges emerged during the process, but the joint work involving the three levels of government enabled the identification of solutions and adaptations within the outlined dynamics. The primary required adjustment was the use of the guidance for certification proposed by the WHO,^8^ as the main reference for updating the Brazilian guide,^9^ instead of the certification model proposed by PAHO,^7^ which recommended the certification of dual EMTCT - HIV and syphilis -, in addition to including hepatitis B and Chagas disease. This adjustment was necessary to allow separate certification for HIV or syphilis, and the inclusion of the tiers of progress towards EMTCT. The adjustment aimed to increase the number of candidate municipalities, as it was necessary to encourage them to prioritize MTCT on their agendas. Despite geographical challenges, local specificities and determining factors related to social context, the process enabled improvements in surveillance and care procedures in the municipalities visited.

One barrier found in the experience was the challenge of measuring the indicator of coverage for pregnant women who underwent at least one HIV and/or syphilis test during prenatal care, given that there is no single national data source for its verification. It could be seen that adherence to the Ministry of Health’s electronic medical record system, which allows this information to be recorded, is heterogeneous across the country. It was also noted that a significant number of the candidate municipalities performed rapid testing, but did not systematically record this procedure. In addition, there was a gap in collecting this data within the private healthcare network. Therefore, the alternative found was to allow the systematic analysis of medical records, based on a representative random sample of live birth population in the years evaluated, namely: the medical records from the last full year for impact targets; and the last two full years for process targets. This measure was important and further motivated the municipalities to participate in the certification process for the elimination of MTCT of HIV and/or syphilis.

Prenatal care is crucial for achieving favorable pregnancy outcomes; its quality is related to the availability of supplies and the establishment of care routines.^11^ ^),(^ ^14^ Some strategies can help improve prenatal testing, including the implementation of the dual HIV/syphilis rapid diagnostic test, which is considered cost-saving in several countries.^17^ ^),(^ ^18^ Another important strategy involves sensitizing primary care professionals in order to improve the notifications of cases and procedures performed during care, making them an integral part of the surveillance system.^19^

An opportunity in conducting this process was the face-to-face visits to the municipalities, which provided the exchange of experiences and knowledge, mobilization of local teams, identification of strengths and weaknesses in the processes developed in the locations, as well as encouraging the use of national information systems and the electronic medical record in primary health care. The certification process is a strategy for mobilization and continuous education. The indicators are monitored across the three levels of management annually, encouraging empowerment through recognition of the joint effort made. The stages of the process have strengthened the integration between surveillance and health care, intersectoral collaboration, intergovernmental management and social control in tackling EMTCT. All of these elements contribute to a continuous learning methodology and local improvement, operating as a potent mechanism for inducing the achievement of process and impact targets, towards the EMTCT of HIV and syphilis.^20^ ^),(^ ^21^

By including the axis of “human rights, gender equality and community participation”, the certification process has promoted the recognition of municipal experiences in prevention, diagnosis and treatment, in the context of populations facing vulnerability and inequity in access to these services. Furthermore, it highlighted the need to promote community participation in instances of social control, such as health councils and ombudsman offices.^9^ This is a continuous and prioritized movement on the national agenda, given that Brazil is a PAHO signatory country to the EMTCT Plus initiative and, more recently, in the elimination of diseases - those amenable to elimination - in the Americas. In this sense, a plenary meeting of the tripartite committee, a forum comprising the Minister of Health and the presidents of CONASS and CONASEMS, resulted in the National Pact for the Elimination of Mother-to-Child Transmission of HIV, Syphilis, Hepatitis B and Chagas Disease as a Public Health Problem by 2030.^22^ Therefore, the Guide for Certification of the Elimination of Mother-to-Child Transmission of HIV and/or Syphilis is being updated to include hepatitis B and Chagas disease in the subnational certification of these health conditions.

The experience with the certification process of EMTCT of HIV and/or syphilis in 2022 was important in mobilizing the municipalities that engaged in its initiatives, worked to improve the quality of care and emerging as the principal proponents in the process. The participation of the three levels of government - federal, state and municipal - civil society and the scientific community showed the strength of tripartite governance, especially in a country with continental dimensions such as Brazil. For 2023, the whole process is being repeated with the anticipation of over 100 municipalities joining. The experience of the subnational certification of the elimination of MTCT of HIV and/or syphilis showed that, beyond the outcomes achieved, the methodologies employed were of paramount significance, providing change in service routines and better integration of surveillance with health care.
